# Between Shell Shock and PTSD? ‘Accident Neurosis’ and Its Sequelae in Post-War Britain

**DOI:** 10.1093/shm/hkx118

**Published:** 2018-02-01

**Authors:** Ryan Ross

**Keywords:** accident neurosis, post-concussional syndrome, shell shock, PTSD, trauma

## Abstract

This article focuses on the concept of ‘accident neurosis’, popularised by neurologist Henry Miller in studies published in 1961. It aims to realise two goals. First, it introduces Miller’s concept of accident neurosis to the broader history of trauma—to a field, that is, more preoccupied with military traumata and clear-cut psychiatric aetiologies. Secondly, I use Miller’s studies, and the considerable legacy they created, to reflect on how historians of trauma construct historical narratives, asking whether there is sufficient appreciation of the ways in which events seem to leak into or retroactively animate one another.

## Introduction

‘Litigation neurosis’, ‘post-concussional syndrome’ and ‘compensation-itis’ were the various labels employed by twentieth-century physicians to the cluster of traumatic sequelae that followed traffic and industrial accidents—symptoms which ranged from headaches, dizziness and mood changes, to restlessness, insomnia, depression, anxiety, gastric disturbance, social withdrawal or lack of appetite, libido or concentration.^1^ Such symptoms, contemporaries observed, bore an inconstant relationship to the severity or site of an injury, only ever occurred in situations where compensation was payable and did not improve until a monetary claim had been settled.^2^ The thinking ran that post-accident symptoms were exaggerated or prolonged by the sufferer through an unconscious wish for financial recompense—a desire, doctors lamented, that was immanent to the systems of compensation and insurance that emerged in the twentieth century.^3^

Indeed, such was the conclusion of neurologist Henry Miller in a study that formed the basis of the two Milroy Lectures he gave at the Royal College of Physicians of London in February 1961.^4^ Often identified as the first to publish a follow-up study of medico-legal claimants, Miller used the Lectures to provide an extensive statistical breakdown of the few symptoms he had encountered in his patients following the cessation of their claim.^5^ Although he opted for his own terminology (‘accident neurosis’), Miller’s conclusion was fairly typical of the literature on accident-related sequelae—that symptoms usually disappeared once a claimant exited the medico-legal process, and that other factors were more important in determining the trajectory of the accident neurosis (e.g. cupidity).^6^ As Miller complained, the typical neurotic was gripped by ‘an unshakeable conviction of unfitness for work’, ‘an absolute refusal to admit to any degree of symptomatic improvement’ and ‘bitter … [complaints] of disabling nervous symptoms lasting many months … for which [the claimant] has never once sought medical treatment’.^7^

Miller was the most influential and well-cited contributor to debates over the traumatic sequelae of accidents in post-war Britain, and one of the purposes of this article is to explain why this was, to provide greater context on a neurological study that implicates various historiographical issues but which has thus far received little widespread attention.^8^ Yet, through my focus on Miller, I aim to realise a more ambitious goal, not merely to rectify a lacuna but also to effect a reorientation in how historians study disorders like accident neurosis, for the focus of this analysis is not the history of occupational health, but the history of trauma.^9^ Although the former may make claim to accident neurosis, this article follows contemporary medicine in locating the disorder alongside a wide range of traumatic sequelae, including traumatic hysteria, neurasthenia, shell shock and railway-spine.^10^ In so doing, this article agitates for a broadening of the historical study of trauma beyond the two disorders that, more often than not, act as its bookends—shell shock and post-traumatic stress disorder (PTSD).^11^

The centrality of these two disorders to the historical study of trauma is now beginning to be questioned.^12^ Yet earlier studies by Ralph Harrington and Tracey Loughran have already warned of the detrimental effect of having PTSD as the expressed or implied end-point of historical research. They argue that it has allowed present-day psychiatric thinking to set the agenda. For example, with respect to the traumatic sequelae of nineteenth-century railway-accidents (dubbed ‘railway-spine’ by contemporaries), Harrington validly criticises the teleology informing certain studies, in which railway-spine has been co-opted into a broader narrative about the rise of psychological theories of trauma. So as to ease comparisons with other traumatic disorders, Harrington shows, railway-spine has been misrepresented by historians as a psychological disorder.^13^ A similar point is made by Loughran in respect of shell shock. She identifies in historical studies of it and the First World War a presentist assumption in which shell shock is regarded as fundamentally psychiatric, with contemporary physiological understandings of traumatic breakdown thereby portrayed as wrong or diversionary. According to Loughran, this stems from an implicit acceptance of present-day thinking on PTSD and the psychiatric aetiology of traumatic disorders. Conceiving of shell shock as a psychological disorder may make the disorder more amenable to genealogies of PTSD, she argues, but it also forecloses other areas of historical research.^14^

The arguments advanced by Harrington and Loughran are, in my view, compelling; they serve as my principal interlocutors in pushing the history of trauma beyond shell shock and PTSD. Indeed, as Loughran explains with respect to the former, it matters the way historians conceptualise past traumatic disorders, as ‘it affects how shell shock [or any other disorder] is fitted into other historical narratives, including the histories of twentieth-century war, medicine, psychiatry, and psychology’.^15^

This article extends this critique one stage further, arguing that interest in PTSD has not only led to what Harrington and Loughran identify as misunderstandings of railway-spine and shell shock, but also a preoccupation with psychiatric and military traumata more broadly.^16^ Hence why certain traumatic disorders sit outside the remit of historical study: consider, for example, why there are so many histories of shell shock and PTSD, but so few on traumatic disorders associated with non-military settings or which possess an ambiguous aetiology (e.g. whiplash or post-concussion syndrome).^17^ Furthermore, it is my contention that the teleology Harrington and Loughran identify has not only created blind-spots in terms of what we include within the historical narrative, but has also shaped how we tell that narrative—encouraging historians to represent the development of medical thinking as a chronology of events, but thereby over-looking how that chronology is upset by those events to retroactively bleed into, catalyse or animate one another.^18^

This brings me back to Henry Miller, accident neurosis and the two-fold task of this article. First, through a study of contemporary medical publications, it aims to introduce Miller’s conceptualisation of accident neurosis to the history of trauma, to a field more preoccupied with clear-cut aetiologies and affinities with PTSD.^19^ My second aim is more philosophical. It concerns Miller’s legacy and the chronologies that historians construct. My argument is that Miller’s 1961 lectures aroused only modest interest in the 1960s. Their popularity came a decade later, as physicians sought to replicate Miller’s study with new groups of claimants. The significance of Miller’s work soon implicated it in multiple debates within the medical press—about post-concussional syndrome, malingering, iatrogenesis—which I want to account for with reference to a changing epistemological context. But I also argue that the Milroy Lectures were, in part, a cause of this intellectual shift—that Miller’s work became popular because of both the impact of the past on the present, in creating momentum, but also the way in which the present over-coded the past, as physicians continuously returned to, and re-animated, the Milroy Lectures in the decades following their publication.

In so doing, this article conceives of the history of trauma not as a chronology, but as a composition of co-extensive layers, that seep into, colour and catalyse one another.^20^ Through this, I aim both to locate Henry Miller’s concept of accident neurosis within the history of trauma, but also encourage a rethink about what models of time and causality historians activate in narrating that history. I develop this argument across the seven remaining sections of this article, the first three of which give an overview of the Milroy Lectures, of Miller’s faith in statistics and of the follow-up study he first reported on in 1965. I then pivot to a commentary on the immediate and subsequent long-term influence of Miller’s study of accident neurosis, drawing attention to the new-found faith in statistics that the Milroy Lectures encouraged and the effects of that on how medics re-read Miller’s work. My conclusion returns to my opening remarks on the history of trauma, and reflects on what historians include within that history and of how they choose to narrate it.

## The Milroy Lectures and the Archetypal Case of Accident Neurosis

The basis of Miller’s 1961 follow-up study derived from a somewhat convoluted collection of medico-legal claimants. He offered general, anecdotal observations on traumatic sequelae based on his experience of examining around 4,000 claimants over a 12-year period. This was complemented with more specific statistical data on the prevalence of neurosis with recourse to two further groups of claimants—200 consecutive patients with accident-related head injuries, whom Miller had personally examined between 1955 and 1957, and a second group of 50 ‘unselected’ claimants who had presented to Miller with ‘disabling nervous symptoms’ during examination and consequent settlement of their case.^21^ The content of the Milroy Lectures stemmed from these three datasets, with qualitative evidence abstracted from the 4,000 claimants, and quantitative findings derived from the groups of 200 and 50 claimants. It was through a study of these three datasets that Miller concluded that cupidity was the principal cause of the prolongation of traumatic sequelae, that nervous symptoms were often elaborated by the claimant’s psychology.

It was with reference to the group of 200 claimants that Miller opened his first lecture. He regarded the group as sufficiently representative for conclusions to be drawn from them, noting that the majority (152) were male, mostly aged between 20 and 60 (the average age was 42), coming almost equally from industrial and traffic accidents (106 from the former; 94 from the latter) and that they came from a wide range of social backgrounds (‘from unskilled labourers to the peerage’).^22^ It was with reference to this group of 200 subjects that Miller quantified the prevalence of neurotic sequelae. He identified 47 of them as having ‘gross and unequivocally neurotic complaints’, a further 22 as suffering from post-concussional syndrome with some neurotic overlay and an additional nine as showing symptoms of endogenous depression; of the remaining 122, Miller found one case of traumatic delirium/schizophrenia following severe head injury, and another 34 with organic impairment of intellect or personality.^23^

The 47 claimants with gross neurotic symptoms were used by Miller to provide another statistical breakdown of the prevalence of traumatic sequelae—out of the 47, he noted, claimants were twice as likely to have suffered injury in industry rather than on the road, and were also twice as likely to be male. It was with reference to the 47 neurotics, furthermore, that Miller introduced one of several statistical inferences that provided the foundation for his lectures. He emphasised what he identified as an inverse relationship of physical injury to the severity of psychiatric symptoms: 31 per cent of patients without skull fracture nevertheless presented with nervous sequelae, whereas only two out of 25 of those with compound skull fractures ever complained of neurotic symptoms (one of whom was a ‘lifelong hypochondriac’ anyway). Indeed, 42 per cent of the patients who were never actually unconscious presented with traumatic sequelae (this was against 14 per cent of all unconscious patients). Meanwhile, 37 per cent patients with post-traumatic amnesia (PTA) of less than 15 minutes developed neurotic sequelae, against just three out of 48 who had PTA of over 72 hours.^24^

Miller’s statistics were of particular note, I want to suggest, and a contrast with the studies published before 1961.^25^ Many of these were similar to the Milroy Lectures in that the author drew upon patients whom he had encountered during medico-legal examination.^26^ Yet there were few statistical studies of the incidence of traumatic sequelae, with most physicians evidencing their argument with qualitative data (anecdotal observations mainly, and case histories where relevant). Miller’s lectures made use of statistics to avoid what he identified as the limitations of earlier research, for his gripe was that there had been few substantive studies published of accident neurosis, with most formed of ‘occasional contributions, often more conspicuous as expressions of opinion than for their factual content’.^27^ Miller’s aim, in the 1961 Milroy Lectures, was to produce a study of sufficient scientific rigour, one that did not rely on subjective analysis or ‘expressions of opinion’. Hence his attraction to statistics, to what he later described as the use of ‘objective parameters as far as possible’.^28^ Statistics were an ‘objective’ means of gaining control over a field that was characterised by terminological slippage and incomparable data.^29^ They allowed Miller to prove connections that had previously been hypothesised but not firmly demonstrated.^30^

Take, for instance, the length of time taken off work, and the relationship between it and the severity of injury. Although an inverse relationship had previously been advanced by physicians, few had provided any substantive evidence in support of it. In his study, however, Miller was able to show a statistical correlation, where the less severe the injury the greater the chance of occupational disablement. So, on average, 31 neurotics had taken six months off work post-accident (despite only nine of this group losing consciousness for longer than an hour). By way of contrast, the average length of disablement was just under four and a half months for patients with compound skull fractures but no neurotic sequelae. Miller made a similar point with respect to post-concussional symptoms, and it was again through the use of statistics that he claimed to be able to demonstrate another inverse relationship between the severity of post-concussional symptoms (as reported by the claimant) and the presence of organic aetiology (as deduced by the physician). Miller undertook a comparison of two datasets, both drawn from the original series of 200 consecutive head injuries. First, he noted that 73 of the 200 patients complained of post-concussional symptoms following closed head injury (e.g. headaches, dizziness, failure to concentrate, etc.). Nine out of 22 patients with compound fractures made similar complaints. Yet none of the compound fractures displayed symptoms indicative of a ‘neurotic superstructure’ (e.g. insomnia, depression, anxiety or self-pity). In contrast, 22 of the 73 patients with closed head injuries complained of neurotic sequelae and, in some cases, that their symptoms were failing to improve or were worsening by the time of their second or third examination.^31^

It was this relationship between injury and psychiatric sequelae that Miller returned to in 1965, in a co-authored paper that focused not on minor but on severe head injuries (that is, where the sufferer had a PTA of over 24 hours’ duration; the average of the sample was 13 days). The findings offered further support to the claim that there was no positive relationship between the severity of head injury and psychiatric injury, and Miller again drew extensively on statistical data to reinforce his point.^32^ Based on follow-up examinations of 92 survivors, drawn from a consecutive group of 100 injured patients examined several years previously, Miller observed that neurotic complaints were fairly infrequent in his sample: only ten of the original 100 had presented with such symptoms, and in only two of the ten had the neurosis been disabling. Indeed, only four out of the 92 who were re-examined complained of neurotic symptoms, two of whom Miller identified as having predisposing personalities. In fact, Miller commented on the relative success of the claimants in recovering from head trauma, with the majority having returned to work, by the time of follow-up, at the same or higher status than they held prior to their accident—only ten out of the 85 adults in Miller’s sample had been totally disabled. A further group of 30 had been demoted by their employer (six of which for epilepsy) but this was for a range of factors, including impaired efficiency but also ‘personal inclination’.^33^

## Objectivity and Predisposition

Miller’s reliance on statistical data, in both 1961 and 1965, points to what I identify as his faith in the importance of ‘objective’ research on accident neurosis. Miller was caustic, for example, of the clinical literature on head injury, bemoaning that it was ‘extensive, inconclusive, contradictory, and based almost entirely on clinical impression’.^34^ He was equally unimpressed with psychiatrists’ aversion to statistical evidence, and complained that there was no objective evidence for the post-concussional syndrome, that it was usually just assumed to exist by physicians (‘sanctified by tradition’).^35^ It was in his pointed comments on predisposition, however, that Miller’s stress on objectivity was most pronounced. He discounted the importance of constitutional factors in the aetiology of post-traumatic symptoms, at least as traditionally conceived by psychiatrists: although he was willing to countenance the effects of a predisposing personality, Miller was sceptical about how they could be deduced, arguing that their assessment was both ‘subjective’ and ‘retrospective’ (a mere ‘clinical impression’).^36^ Instead, he pointed out that only 20 of his group of 47 neurotics—less than half—actually displayed evidence of what psychiatrists would have identified as predisposing behaviour. Indeed, Miller complained that claimants often attempted to deny the prevalence of predisposing factors anyway, and it was unsatisfactory to rely on other sources (e.g., health-records, testimony from general practitioners) as they were produced for different purposes. Miller instead advocated for a different way of understanding predisposition, one that was more social than psychiatric, and which—crucially, he argued—was predicated on a more reliable, accurate means of assessment.

Miller proposed that most of those who developed neurotic symptoms were drawn from either the unskilled or semi-skilled classes, with labourers, cleaners and mineworkers the most prevalent groups in his sample. Indeed, although he acknowledged what other physicians identified as the ‘nature of the accident’ as a possible cause of nervous sequelae, Miller countered that, of the 17 most distressing accidents that his claimants had been involved in, only two of them ever presented with neurosis.^37^ For Miller, low intelligence was a more reliable means of assessing predisposition. Work history was even better—if an individual was often absent from work, changed their job a lot or had been off for a long time following previous minor accidents, then this information, Miller claimed, could be used to accurately judge the claimant’s likelihood of being predisposed to neurosis.^38^

Having dismissed the importance of psychiatric predisposition, and with recourse to the statistical prevalence of certain symptoms in his sample, Miller then outlined in the Milroy Lectures what he characterised as the defining features of accident neurosis. He excluded organic damage as a cause of neurosis (its relationship to psychiatric sequelae was inconstant), and instead identified headache, dizziness when stooping, irritation, inability to concentrate and restlessness as the most common complaints made by claimants. In roughly half of cases, furthermore, the claimant voluntarily complained of sleeplessness, but almost all admitted to insomnia or nightmares when asked. Objective symptoms, Miller further noted, were present in less than 15 per cent of his personal cases (this included tachycardia, tremor or hyperhidrosis). Yet ‘gross dramatisation of symptoms’ was present in over half of claimants, such as in the way they recoiled from ophthalmic examination, groaned excessively when their spinal flexion was tested or else ‘slump[ed] forward with head in hands during consultation, requesting a glass of water’.^39^

Miller moved from quantitative analysis to his own observations in concluding his outline of the archetypal case of traumatic neurosis. For example, the claimant was frequently antagonistic towards the medical examiner, unable to relax and usually late to the examination. If the claimant was accompanied, it was usually with a member of his family (his wife, typically), who then proceeded to do all of the talking, reminding the claimant of any details that he had forgotten (Miller thought it curious that even when the claimant complained of amnesia, he was still able to provide a full account of the accident and what followed). Moreover, the claimant always asserted that his accident was the fault of someone else, an unspecified ‘them’ who were ‘in some vague way identified with the implying organization [sic] or the unknown motorist’.^40^

It was these features, in combination with the inverse relationship between injury and symptoms, that led Miller to argue that it was the medico-legal process that caused the development of traumatic neurosis, that the events that followed the accident in time then folded back upon it, magnifying or elaborating otherwise trivial symptoms: the injured workman was impressed upon by a variety of influences from the moment of his accident, with insurance officials, family members, doctors and solicitors unwittingly colluding to transform the workman’s minor injury into a debilitating one.^41^ Yet Miller wrote of his wish to substantiate his anecdotal evidence with further statistical data, and complained that there was little reliable information on what happened to claimants once their claim was concluded. His solution was to undertake his own follow-up study of 50 claimants whom he had previously examined but had since exited the medico-legal process.

## The Follow-up Study

Miller never specified how his group of 50 follow-up cases were selected, instead focusing on their social composition. Thus, he explained that 41 of the group of 50 were male; that just under half of them (24) were unskilled or semi-skilled workers; that 35 had suffered ‘trivial’ injuries (and in three no injury whatsoever); and that 31 had been injured in industrial accidents and 18 on the road (where the remaining one subject came from was not specified). Miller further noted that, of the 50 cases, 42 had been settled by negotiation, four abandoned or withdrawn, with the remaining four having proceeded to trial (which the claimant then lost). The average delay between accident and settlement was 26 months, and damages ranged from £20 to almost £5,000, with the average payment being £454 (although in some cases this was calculated on the basis of limited liability). The average time between settlement and re-examination in Miller’s sample was just over two years.^42^

Miller’s follow-up study supported the hypothesis that traumatic symptoms allayed after the claimant had exited the medico-legal process: altogether, of the 50 patients whom Miller had examined after the termination of the claim, only two had psychiatric problems disabling them from work (three had psychiatric disorders but still worked). Indeed, 41 out of 45 of those who had been employed prior to their accident had returned to work by the time of Miller’s re-examination, with 45 of the 50 symptom-free (they could only ‘muster … a few trivial residual symptoms’, Miller noted, like nervousness in traffic).^43^ This was in contrast to their emotional state during Miller’s original medico-legal examination, with 36 of the 50 displaying symptoms typical of traumatic sequelae (‘… depression, restless sleep, hypochondriac invalidism, disgruntlement, and self-pity in various proportions’). Indeed, of the 36, Miller identified 21 of them as suffering from some form of hysterical reaction, whilst a further five had developed a phobia.

It was from this discussion of his follow-up claimants that Miller moved, in the second Milroy Lecture, back to his own observations, writing more broadly about the typical neurotic. Indeed, if his first lecture in 1961 was concerned with outlining the statistical data he had collected, his second lecture was tasked with articulating what he identified as the cause of prolonged traumatic sequelae. Miller’s first claim was that accident neurosis affected between a quarter and a third of all those involved in accidents both when the accident was someone’s else’s fault and when it occurred in a setting where compensation was available.^44^ Moreover, those who did develop accident neurosis, Miller argued, were not those predisposed, but those who came from a low ‘social or occupational status’. Yet Miller challenged the view that economic insecurity amongst the lower social classes was a causal factor in accident neurosis, for accidents in non-compensable environments (e.g. on the sports field) did not cause such long-lasting sequelae but must still have had the potential to incapacitate the sufferer. In addition, Miller noted that there was a ‘flood’ of claims under common law despite the introduction of national insurance, and that many claimants insisted that they could not return to work while their case was ongoing (even though this would have been more financially rewarding than unemployment benefit). Instead, Miller postulated that accident neurosis was related to a lack of social responsibility on the part of the claimant, that those claimants with less autonomy in their work, and less of a stake in the fruits of industry, were more likely to develop traumatic sequelae.^45^

This pivoted to a broader social commentary by Miller. It concerned malingering, which he identified in the Milroy Lectures as rare. Yet it was far rarer for the disorder to be diagnosed by physicians, Miller complained, even though fraudulent claims were countenanced—indeed, often freely admitted—by trade-union officials, insurance agents and lawyers. He further added, in his 1961 lectures, that although he struggled to properly identify malingering in neurotic claimants, he was far surer of the presence of exaggeration: despite being unable to decide whether it was unconscious or conscious, Miller was firmly of the view that both were just as bad and that attempting to condone self-deception was ‘strange equity and stranger logic’.^46^ These views would harden in later publications, with Miller commenting in both 1966 and 1972 that unconscious exaggeration and outright fraud were indistinguishable, that both were forms of malingering caused by western systems of welfare and the dissemination of medical knowledge amongst the lay public (especially knowledge of head injury, which, according to Miller, made concussional symptoms easier to simulate than something like spinal cord injuries).^47^ In summary, Miller identified the medico-legal process as responsible for the problem of accident neurosis, and castigated the medical profession for not properly grasping the problem.

## The Milroy Lectures in the 1980s

It would, I think, be incorrect to argue that the Milroy Lectures received little exposure in the decade following their publication, for there were several responses published in the medical press, including supportive editorials in the *British Medical Journal* and *The Lancet* in 1961.^48^ Miller’s research was also discussed at a meeting of the Royal Society of Medicine (RSM) in 1965.^49^ However, it was not until the 1970s and 1980s that Miller’s work attracted its widest audience. Moreover, the nature of this popularity differed substantively from earlier responses—it was more focused, that is, on the methodology of follow-up studies, on how research into neurotics might be better realised once Miller’s approach was modified. The ensuing debate illustrates what I think of as a temporal immanence to the Milroy Lectures, of how they both shaped and were shaped by the studies that followed—by attempting to replicate, compare and contest his earlier findings, Miller’s detractors were both influenced by (and determined to avoid the biases of) the Milroy Lectures; but, simultaneously, by attempting to replicate, compare and contest the Milroy Lectures, Miller’s detractors helped install him as progenitor of a new if imperfect way of studying the post-concussional syndrome. My aim, in the remainder of this article, is to show how this came about.

The earliest published responses to Miller were mainly written by GPs, and were broadly supportive of many of the points raised in the Milroy Lectures.^50^ They included a plethora of solutions and demands, ranging from the greater provision of medical officers in industry, to a renewed willingness by doctors to intervene in cases of accident neurosis.^51^ In addition, some advocated for the creation of a government-led inquiry, Royal Commission or joint committee of the medical and legal professions, in the hope that this could devise new ways of reducing delays in the medico-legal process.^52^ All concurred, however, that treatment was useless until a claim had been concluded. Indeed, an editorial in the *British Medical Journal* praised Miller’s intervention, agreeing that accident neurosis was a reflection of ‘industrial morale’, of the ‘us-vs-them’ thinking that struggled to be contained by the Welfare State.^53^*The Lancet* offered a more guarded assessment, pondering whether self-deception should be thought of as a symptom of illness when the patient could not consciously control their will.^54^ This was, however, a minority view in 1961. Initially, most of the published responses were supportive of Miller’s findings, and praised him for introducing the concept of accident neurosis to a broader audience.^55^

It was not until after 1965 that the first explicit challenges to Miller’s work began to appear, one of which came from neurosurgeon A. R. Taylor.^56^ He regarded post-concussive sequelae as organically caused, a product of brain damage or increased intracranial blood flow.^57^ His challenge to the Milroy Lectures was three-fold, and focused on certain assumptions that Miller had made in his follow-up study. First, Taylor argued that a skull fracture might not be as bad as initially thought: it meant that the brain had been able to move inside the cranium, for the skull to absorb the blow and not the brain. According to Taylor, a fracture was evidence that the skull had protected the brain, and, as such, it was incorrect of Miller to use it to evidence severe head trauma. Secondly, although Taylor agreed with Miller that social responsibility was important in the development of neurosis, he disputed whether it was fair to compare sports with traffic incidents, since the latter typically occurred at higher velocity. Finally, Taylor argued that follow-up studies of the head injured, from both the USA and Germany, showed that recovery did not always allay once payment had been made, that Miller’s stress on cupidity was misplaced.^58^

A second challenge to Miller came from neuropsychiatrist W. A. Lishman at a meeting of the RSM in 1965. Both Miller and Lishman spoke at the meeting, with the former taking it as an opportunity to repeat the findings from the Milroy Lectures. Lishman, however, countered that post-concussive sequelae were probably related to brain damage. His argument, furthermore, relied not on logical deductions, as with Taylor, but some preliminary statistics of his own. These he had derived from an analysis of 670 patient files from the Oxford Military Hospital for Head Injuries.^59^ Lishman’s study of these records was highly qualified—he admitted that he had difficulty identifying one clear relationship between head injury and psychiatric disability, and, indeed, was unable to fathom precisely what psychiatric disability constituted (it was defined in different ways in the medical literature). Furthermore, both he and Miller were studying different degrees of head injury, with Lishman focusing on more severe, penetrating damage (and more severe psychiatric symptoms, too).^60^ Nevertheless, Lishman argued that his research indicated a quantifiable relationship between brain damage and behavioural, affective or intellectual changes observed of the patient—Lishman’s statistics demonstrated a correlation between the length of PTA and psychiatric disability, for example, as well as between sensory/motor defect and psychiatric disability. The follow-up study, Lishman also claimed, suggested a correlation between the site of head injury and psychiatric disability, although further research was needed to develop this fully.^61^

Lishman would renew his thesis throughout the 1970s, as he refined his analysis of what he had found in the Oxford follow-up studies. He was not alone in seeking to challenge the Milroy Lectures, however. There existed a broader backlash against Miller in the 1970s, a consequence of unresolved medical debates—the profession had been unable to agree on the precise aetiology of the post-concussional syndrome, with debate ongoing as to how to best identify and treat the concussed patient.^62^ Miller’s identification with one side of the debate, encouraged by the pugnacity of the Milroy Lectures, ensured that his work remained influential in the years after 1961: he was the figure-head of what was later described as an ‘extreme’ position by psychiatrist W. H. Trethowan.^63^ Writing in 1970, for instance, Trethowan argued that although Miller was right to highlight the role of compensation in the aetiology of traumatic sequelae, he had overlooked the evidence for the post-concussional syndrome’s organic cause.^64^ A not dissimilar point was made by orthopaedic surgeon R. Gruneberg in the same year, who complained about the imprecision of the term ‘accident neurosis’, and of the prejudice that could enter medical diagnosis through its use.^65^ Later, in 1973, Lishman countered Miller’s claim about the inverse relationship between symptoms and the severity of an injury, arguing that although compensation could cause a functional overlay, this rested on top of organic damage to the brain.^66^

The force with which Miller had entered the debate ensured that his work would be picked over throughout the 1970s, and with great vigour. His only respite was in medical debates over malingering, where physicians, animated by what Richard Asher had earlier labelled the ‘borderlands of malingering’, identified self-deception as a growing medical problem, evident in disorders like anorexia, Munchausen’s, self-harm and accident neurosis.^67^ Yet such debates were small relative to the grander contest over the cause of post-concussional syndrome. In fact, in the decade after 1965, a consensus formed amongst neurologists and psychiatrists that concussive sequelae were causally related to brain damage, even if the patient’s psychology elaborated the symptoms.^68^ Indeed, at the 1965 meeting of the RSM, at which both Miller and Lishman presented their papers, one speaker commented favourably of this approach. Psychologist Oliver Zangwill, responding to both Miller and Lishman, argued that the former had placed too great a stress on the psychological basis of the post-concussional syndrome. Citing his wartime experience, Zangwill commented on the high number of patients he had examined with mild but persistent intellectual impairment.^69^ But Zangwill also issued a note of caution concerning the use of statistics in the study of the post-concussional syndrome, commenting that large-scale statistical studies like Lishman’s should not prevent a more fine-grained study of location in the development of psychiatric disability.^70^

It was one of a number of warnings made against the use of statistical inference in studying the post-concussional syndrome. They largely went unheeded. Although his view was coming under attack by other doctors, Miller’s influence with the legal profession was considerable (it was claimed that the Milroy Lectures were regularly cited in court).^71^ This incited many of the studies published in the 1970s. But it also encouraged Miller’s detractors to change strategy, for they had thus far been able to criticise but not fully refute the Milroy Lectures. Taylor had attempted to unpick them through logical deduction, but his research was scarcely cited. Meanwhile, critics complained that it was Miller’s statistics that were the most frequently referenced aspects of his accident neurosis study.^72^ Consequently, many physicians began to argue that the Milroy Lectures could only be countered by undertaking fresh follow-up studies, to compile new statistics on head injury.^73^ In effect, Miller’s now decade-old research was transforming how physicians studied the post-concussional syndrome.^74^

The Milroy Lectures were to have an additional effect on the medical literature, I want to argue, for the greater use of statistical studies worked not merely to identify the Milroy Lectures as originator of a new approach to research, but also encouraged comparisons, re-readings and critique of Miller’s findings. However paradoxically, Miller had instigated a change in how the post-concussional syndrome was studied, but this then led, as a consequence, to repeated re-investigation of Miller’s methodology and conclusions. Research published post-Miller, therefore, had not only to produce some figures from following-up with claimants, but also had to reflect carefully on how these had been produced, to prevent them from perpetuating the early biases of the Milroy Lectures: Miller had reconfigured the study of traumatic sequelae, but, in so doing, had invited further scrutiny and reading of his work. This is best illustrated with reference to the publications of neurologist Reginald Kelly.

## Reginald Kelly and the ‘Post-Traumatic Syndrome’

Kelly was, of all the contributors to the medical debate in the 1970s, the most critical of Miller, and especially of his methodology, identifying the Milroy Lectures as the cause of a misguided approach to traumatic sequelae. Indeed, whilst Kelly had no complaint with the use of statistics *per se*, he contended that Miller’s figures presented a skewed picture of the neurotic patient, writing that if the stats ‘contradict what has been clinically obvious’, then the ‘source of the figures and the prejudices of the statistician’ should be questioned.^75^ He claimed, for example, that Miller’s sample was comprised of claimants referred to him by insurance companies, and therefore represented those whom the insurers thought that they could challenge (the ‘genuine’ suffers would have already had their cases settled, as would those who recovered spontaneously prior to settlement). Moreover, Kelly pointed out that insurance cases could drag on for many months, and the most severely injured were less likely to be pestered by insurance officials anyway: Miller, in other words, had been studying the most hardened neuroses.^76^ Kelly further complained that Miller was simply reporting, and in retrospect, on someone else’s ‘mismanagement’ of the concussed patient, rather than observing the patient in a prospective study, or, indeed, seeking to intervene in the patient’s treatment.

This was one of several counter-proposals advanced by Kelly during the 1970s: for instance, he would argue that malingering was possible but rare in many cases, and that it was unclear why neurotics exaggerated their disorder when, according to Miller, they did not gain financially from doing so.^77^ Yet it was the role of statistics in the Milroy Lectures that Kelly kept returning to. One of Kelly’s gripes was that Miller privileged numbers over clinical observation, complaining that although it was now popular to denigrate ‘anecdotal comments in papers … many of our colleagues have forgotten that we still have much to learn from the patient if we can retain the ability to step out of our scientific ivory tower’.^78^

One of his first publications on the subject, published in 1972, was explicit in wishing to replicate and thereby test Miller’s statistical study, but with a broader range of follow-up patients (i.e. not just those referred from the insurance company). Kelly derived the bulk of his subjects (112) from referrals from other physicians, and a smaller group (40) from insurers. He noted that the 112 referrals had an average recovery-time of three months, versus 14 months in Miller’s sample and 19 months in the insurance sample. Moreover, although he admitted that neurologists did not see all cases of head injury, Kelly argued that his sample showed neurotic symptoms, as defined by Miller, as more common in the referred patients than the medico-legal claimants (75 per cent of the former, against 65 per cent from the latter). He also claimed that his statistics demonstrated that most neurotic symptoms allayed with proper treatment and before settlement (78 out of the 84 patients, against only 6 out of the 26 medico-legal claimants).^79^

These findings were used by Kelly to buttress his earlier critique of Miller’s methodology.^80^ Indeed, Kelly was thus able to dispute one of the cornerstones of the Milroy Lectures, the supposed inverse relationship between injury and the severity of neurosis. Although he accepted its existence, Kelly questioned the extent of the relationship, claiming that almost half of his severely concussed patients developed neurosis, in contrast to Miller’s more modest findings. Those without emotional symptoms, Kelly concluded, must have been seriously injured to have been unable to remember their trauma. Kelly’s sample also contained a significant number (62) of the managerial/professional classes (including a ‘well-known, successful barrister’). As many as 44 of them, he noted, had neurosis, and were probably so well represented in his sample owing to their intelligence and gumption (i.e. they were less likely to accept that their symptoms would allay naturally, and would seek to be referred to a specialist much more firmly than lower socio-economic groups). Finally, Kelly pointed out that his sample contained 34 non-compensable injuries (sports injuries, accidents-at-home), 24 of whom nevertheless developed neurosis.^81^ The crux of Kelly’s argument was that iatrogenesis was the primary cause of neurosis, and was encouraged by physicians denying the patients’ genuine symptoms. Because the neurotic’s symptoms were not properly believed, nor properly treated or explained, they persisted for longer than normal.^82^ This would be apparent, Kelly averred, were doctors willing to break away from their reliance on Miller’s statistics.

Kelly reiterated this message in the late 1970s and early 1980s, complaining in 1979 that the medical and legal literature was still gripped by certain myths (‘… solemnly enunciate[d] … without recourse to scientific observation or authoritative studies’), principal of which was that the post-concussional syndrome (or ‘post-traumatic syndrome’, as he preferred) was, despite evidence to the contrary, psychologically-caused.^83^ Indeed, in a small follow-up study published in 1981, Kelly attempted to upend the ‘myth’, promulgated by Miller, that patients always recovered post-settlement—Kelly wanted, he explained, to find ‘reliable evidence’ for something that was ‘often accepted without question in the courts, and on which many final settlements have been based’.^84^ His hope was that his research would silence those propounding ‘prejudices rather than the results of observations based on scientifically collected data’.^85^

As far as I am aware, Miller never responded to Kelly in print, to this or any other article of the latter—in fact, Miller stopped contributing to the subject of accident neurosis after the 1960s, his writings thereafter more focused on medical education, ethics or medicine’s relationship with society.^86^ Yet Kelly’s critique of Miller was significant, for it continued to invite re-readings and comparisons with the Milroy Lectures but now allowing a wider group of physicians to undertake their own follow-up studies.

Two studies, published towards the end of the 1970s, illustrate this new trend.^87^ Both were authored by William H. Rutherford and co-authored by John D. Merrett and John R. McDonald, all three of whom were based at institutions in Belfast. They reported on two follow-up studies of concussed patients, undertaken at six weeks and then one year after discharge from hospital. Their findings were relatively banal, in that they can be easily accommodated within what I have described as the medical consensus over the post-concussional syndrome: Rutherford and colleagues argued that many concussive symptoms were either organically or psychologically caused, and could be worsened by litigation or a sense of grievance. What was novel about this study, however, was the professional backgrounds of the authors—unlike Miller, Kelly, Lishman and Taylor, Rutherford was a trauma surgeon, and Merrett and McDonald were statisticians. Granted, they were not the first non-neurologists to undertake a follow-up study of traumatic neurosis, but they were the beginning of what I identify as a new trend ushered in by the use of quantitative research, one that sundered neurologists from the study of traumatic neurosis. Because statistics, relative to other forms of neurological or psychological assessment, did not require unique access to the claimant, nor recourse to sophisticated equipment, follow-up studies could be undertaken with comparative ease by non-neurologists. Symptoms could be recorded via standardised questionnaires or with reference to predetermined lists of questions (about employment, marital status, etc.). What was once the exclusive domain of neurologists (or, occasionally, psychiatrists) was now open to other specialists.^88^ The medico-legal claimant had been extricated from the claims process, and was now open to investigation by physicians of various types of specialism.

That Miller was the primary cause of this change is testified by the numerous references to the Milroy Lectures within the new statistical studies: these, as with Kelly’s research, cited Miller as originator of a new approach to studying post-concussional syndrome (or else, at the very least, identified him as one of the pillars of the debate).^89^ The decoupling of traumatic neurosis from neurological research was helped along, furthermore, by developments in medical thinking, with physicians beginning to argue more forcefully that accident neurosis and post-concussional syndrome were two separate disorders (or were simply the same disorder just viewed at different stages in the patient’s recovery). The effect was to separate accident neurosis from post-concussive sequelae, with many neurologists electing to focus on the latter, and principally with reference to severe head injuries. The field was thus open for other specialists to study neurotic sequelae.

The follow-up studies published by non-neurologists during the 1980s did not blunt Miller’s influence, however. In fact, they swelled the number of references to him, continuing to entrench the Milroy Lectures as both progenitor and point-of-comparison for later studies. For example, orthopaedic surgeon J. E. Woodyard published two studies of traumatic sequelae in 1980, studying a mixture of claimants that primarily included injuries to the cervical spine. Methodologically, these studies were fairly unexceptional—they were both part of a retrospective analysis of 584 medico-legal claimants who had been referred to Woodyard from various sources. But the primary influence on his research was Miller. Thus, for the purposes of his study, Woodyard reviewed the medico-legal reports he had earlier written for each claimant, identifying symptoms and social characteristics, and in a very similar way to what Miller did in the Milroy Lectures. Indeed, Woodyard reached a very similar conclusion to Miller—that physicians were unwilling to diagnose malingering, and that the state, by mishandling the rehabilitation of the claimant, was encouraging a culture of welfare-dependency.^90^ Facilitated by the new trend for statistical study, Woodyard would undertake his own follow-up study two years later, focusing on 52 claimants with trivial injuries who were initially diagnosed with compensation neurosis. Yet Miller still remained influential, acting as a comparison for Woodyard’s group of claimants.^91^ What interests me is less the nature of Woodyard’s complaint about Miller, than that the latter was still acting as a comparator, now 21 years after the publication of the Milroy Lectures. It was one of several occasions when Miller was cited throughout the 1980s, and not just by orthopaedic surgeons.

Following the emergence of statistical follow-up studies in this decade, a greater number of psychiatrists now entered the medical debate, undertaking their own studies often by simply counting the incidence of symptoms and/or the length of time it took the claimant to return to work.^92^ This was the approach of psychiatrists Michael J. Tarsh and Claire Royston, who published their own findings in 1985, having undertaken interviews with 35 claimants who were previously referred by their solicitor for medico-legal assessment. As with Woodyard’s study, Miller’s work was cited first in their study, albeit with some criticism (‘… the first and most influential study … which has held sway in the courts since its publication in 1961, in spite of subsequent work that refutes it’).^93^ Yet the Milroy Lectures again served as point-of-comparison for their own findings (e.g. in terms of the social class of the claimants).

That, owing to Miller, more specialisms were now involved in the quantitative study of traumatic sequelae did not, however, prevent neurologists or neurosurgeons from continuing to contribute to the debate. Many still did.^94^ Discussion, though, was now conducted between a much broader range of voices than before, and with a greater collection of patients. Indeed, this broadening of debate began to produce its own problems. Many physicians, especially psychiatrists, sought to summarise medical debate in articles published during the 1980s, and whilst all explained that the Milroy Lectures had now lost all influence, at least within medical circles, they lamented the diffusion of methodological approaches it had spawned—published studies had since used samples that varied greatly in size, lacked control groups, had categorised symptoms irregularly and had followed-up with patients in various ways and at different stages of recovery.^95^ This had produced a set of data that had arrived a similar conclusions, but which was otherwise incomparable. Greater standardisation was needed, it was argued.^96^ Such complaints informed many of the studies published during the 1990s, principally of whiplash neck injuries (which were more common following the introduction of seat-belts). In a similar vein, the neurologists now studying severe head injury sought to introduce standardised criteria for medical assessment (e.g. the Glasgow Coma Scale). Yet in the literature published in this decade, references to Miller were surprisingly resilient.^97^ The Milroy Lectures were no longer picked over in detail, with references to Miller usually just brief acknowledgements.^98^ However, Miller continued to be identified as chief advocate for the view that post-concussional syndrome and/or accident neurosis was psychologically-caused, or that cupidity was a common motive for medico-legal claimants.^99^ The legacy of the Milroy Lectures, in other words, was considerable, shaping studies for decades thereafter, and retroactively installing Miller as progenitor of this new research agenda.

## Conclusion

Ruth Leys observes that the medical literature on traumatic disorders was highly disparate prior to the establishment of PTSD in American psychiatry in 1980, a variety that has since been ‘obscured by the [post-PTSD] effort to integrate the field’.^100^ Leys was referring to the actions of clinicians, yet her comments should also invite reflection by historians. As the work of Harrington and Loughran suggests, it is important to ask whether the preoccupation with present-day psychiatric thinking—symbolised by the representations of shell shock, and a teleological interest in PTSD—has skewed the historical gaze, privileging clear-cut traumatic disorders over those with ambiguous aetiologies, whilst encouraging an intense focus on military traumata to the relative neglect of disorders like whiplash and accident neurosis, or of rape trauma, Stockholm and battered child syndromes. One task of this article is to encourage rectification of this oversight. Yet I also sought to go one stage further, for if the teleology operating within the history of trauma is to be more fully considered, then it is also worthwhile to reflect on how that history is narrated. As my discussion of accident neurosis illustrates, physicians repeatedly returned to earlier research to inform present-day research, but reconfigured that earlier research in so doing. Indeed, through the medium of the medical journal, physicians were seemingly able to move backwards in time, a process that suggests less a linear chronology of neatly demarcated events than a succession of temporal layers that retroactively bleed into or animate one another. There should be a space within histories of trauma to register these seemingly nonlinear movements, both for studying a disorder and also the effects of its historicization. Indeed, by doing so, perhaps histories of trauma could come to resemble their object of study—they could be characterised, that is, by haunting events, feedback-loops and repetition.^101^

## Footnotes

1 E.g. T. A. Ross, *An Enquiry into Prognosis in the Neuroses* (Cambridge: Cambridge University Press, 1936), 87.

2 E.g. Jeffrey Ramsay, ‘Nervous Disorder After Injury’, *British Medical Journal*, 1939, 2, 386.; and Lindesay W. Neustatter, ‘Psychiatric Disorders in Compensation Cases’, *The Medico-Legal Journal*, 1962, 30, 164.

3 For background on how physicians came to assess compensation claims, and the psychological theories mobilised in so doing, see Roger Cooter, ‘Malingering in Modernity: Psychological Scripts and Adversarial Encounters During the First World War’ in Roger Cooter, Mark Harrison and Steve Sturdy (eds), *War, Medicine and Modernity* (Sutton: Stroud, 1998), 125–48.

4 The Milroy Lectures were published in the *British Medical Journal* in April 1961, and became the subject of editorials in *The Times* and *The Lancet* later that year. See Henry Miller, ‘Accident Neurosis: Lecture I’, *British Medical Journal*, 1961, 1, 919–25; Henry Miller, ‘Accident Neurosis: Lecture II’, *British Medical Journal*, 1961, 1, 992–8; no author, ‘Compensation for Cupidity?’, *The Lancet*, 1961, 277, 1099–100; and no author, ‘Accident Neurosis’, *The Times*, 10 April 1961, 11.

5 Miller (b. 1913; d. 1976) qualified from Newcastle College of Medicine in 1931, thereafter working in Newcastle, then briefly at Johns Hopkins and Great Ormond Street Hospital. He worked under Charles Symonds during the War, and later underwent training at Queen Square before returning to Newcastle in 1947. He became Reader in Neurology in 1961 and then Dean of Medicine at the University of Newcastle upon Tyne a few years after its establishment (Newcastle Medical School had been attached to the University of Durham prior to 1963). Miller became Vice-Chancellor of the University in 1968, a position he held until his death in 1976. Miller’s obituaries are replete with references to his big personality, unique sense of humour and the delight he took in his status as a provocateur. See JNW, ‘H G Miller’, *British Medical Journal*, 1976, 2, 591; no author, ‘Dr Henry Miller: A Distinguished Neurologist’, *The Times*, 27 August 1976, 14; and the compendium of reminiscences and biographies contained in Stephen Lock and Heather Windle (eds), *Remembering Henry* (Tavistock Square, London: British Medical Association, 1977).

6 Miller would later state that he preferred the term ‘traumatic neurosis’ to ‘accident neurosis’ in Henry Miller, ‘Medicolegal Aspects of Head Injury’, *Current Medicine and Drugs*, 1967, 7, 6.

7 Miller, ‘Accident Neurosis: Lecture I’, 922.

8 So far as I am aware, Miller’s concept of accident neurosis has not been the subject of any substantive historical study. For broader histories of related traumatic sequelae stemming from industrial accidents in the twentieth century (or railway accidents in the nineteenth century), see Karl Figlio, ‘How Does Illness Mediate Social Relations? Workmen’s Compensation and Medico-Legal Practices, 1890–1940’, in Peter Wright and Andrew Treacher (eds), *The Problem of Medical Knowledge: Examining the Social Construction of Medicine* (Edinburgh: Edinburgh University Press, 1982), 174–224; Rhodri Hayward, *The Transformation of the Psyche in British Primary Care, 1880–1970* (London: Bloomsbury, 2014), ch. 2; Peter Leese, *Shell Shock: Traumatic Neurosis and the British Soldiers of the First World War* (New York: Palgrave Macmillan, 2002), ch. 2; and Danuta Mendelson, *The Interfaces of Medicine and Law: The History of the Liability for Negligently Caused Psychiatric Injury (Nervous Shock)* (Dartmouth: Ashgate, 1998), esp. ch. 3. For historical overviews of industrial accidents, see Roger Cooter and Bill Luckin, ‘Accidents in History: An Introduction’, in Roger Cooter and Bill Luckin (eds), *Accidents in History: Injuries, Fatalities and Social Relations* (Amsterdam and Atlanta, GA: Rodopi, 1997), 1–16; Karl Figlio, ‘What is an Accident?’, in Paul Weindling (ed.), *The Social History of Occupational Health* (London: Croom Helm, 1986), 180–206; and John C. Burnham, *Accident Prone: A History of Technology, Psychology, and Misfits of the Machine Age* (Chicago and London: University of Chicago Press, 2009).

9 A similar approach is taken in Edgar Jones and Simon Wessely, ‘A Paradigm Shift in the Conceptualization of Psychological Trauma in the 20th Century’, *Journal of Anxiety Disorder*, 2007, 1, 164–75; and Michael R. Trimble, *Post-Traumatic Neurosis: From Railway Spine to the Whiplash* (Oxford: Wiley-Blackwell, 1981).

10 Furthermore, although accident neurosis was widely debated in twentieth-century medical and legal circles, I have encountered few references to the concept within published sources relating to industrial medicine, nor am I aware of any substantive discussions of it by trade unions.

11 The use of shell shock and PTSD as beginning and end of the historical narrative is just as prevalent in studies that elide them together (e.g. as different names for the same universal response to trauma) as they are in historical studies that acknowledge the differences between them, such as Edgar Jones and Simon Wessely, *Shell Shock to PTSD: Military Psychiatry from 1900 to the Gulf War* (Hove and New York: Psychology Press, 2005). Indeed, shell shock and PTSD remain central to easily the most influential genealogies of the latter: Allan Young, *The Harmony of Illusions* (Princeton, NJ: Princeton University Press, 1997). Young’s recent work has, however, sought to move away from what he calls ‘unilinear chronologies’. C.f., Allan Young, ‘Resilience For All By The Year 20—’, in David Cantor and Edmund Ramsden (eds), *Stress, Shock, and Adaptation in the Twentieth Century* (Rochester: University of Rochester Press, 2014), 3–95.

12 E.g. in the broader critique of the Euro-centrism of historical studies of trauma in Mark Micale, ‘Toward a Global History of Trauma’, in Jason Crouthamel and Peter Leese (eds), *Psychological Trauma and the Legacies of the First World War* (Basingstoke: Palgrave Macmillan, 2017), esp. 291–3.

13 Ralph Harrington, ‘On the Tracks of Trauma: Railway Spine Reconsidered’, *Social History of Medicine*, 2003, 16, 209–23.

14 Tracey Loughran, ‘Shell Shock, Trauma, and the First World War: The Making of a Diagnosis and Its Histories’, *Journal of the History of Medicine and Allied Sciences*, 2012, 67, 108–13. See also Tracey Loughran, *Shell-Shock and Medical Culture in the First World War* (Cambridge: Cambridge University Press, 2017), 10–15.

15 Loughran, ‘Shell Shock, Trauma, and the First World War’, 107.

16 Although beyond the remit of this article, it may also be argued that the historical interest in PTSD has led to an excessive interest in post-war American psychiatry, and a consequent diminution in the appreciation of national context, as suggested in Sophie Delaporte, ‘Making Trauma Visible’, in Peter Leese and Jason Crouthamel, *Traumatic Memories of the Second World War and After* (Basingstoke: Palgrave Macmillan, 2016), 23–46.

17 For one of the few histories of whiplash, see Trimble, *Post-Traumatic Neurosis*. Concussive sequelae are becoming better integrated into the historical field, though there is still much work to do. For a promising start, see Edgar Jones, Nicola T. Fear, and Simon Wessely, ‘Shell Shock and Mild Traumatic Brain Injury: A Historical Review’, *American Journal of Psychiatry*, 2007, 164, 1641–5. More generally, on how certain traumatic disorders have attracted historical interest, whilst others have not, see the ‘heterogeneity thesis’ advanced in Allan Young and Naomi Breslau, ‘What Is PTSD? The Heterogeneity Thesis’, in Devon E. Hinton and Byron J. Good (eds), *Culture and PTSD: Trauma in Global and Historical Perspective* (Philadelphia, PA: University of Pennsylvania Press, 2015), esp. 147–50. I am grateful to Allan Young for directing me to this.

18 I am here influenced by the discussion of how causality can be thought of more widely (e.g. in terms of feedback-loops) in Manuel DeLanda, ‘Emergence Causality and Realism’, in Levi Bryant, Nick Srnicek and Graham Harman (eds), *The Speculative Turn: Continental Materialism and Realism* (Victoria, Australia: re. press, 2011), 381–92.

19 The literature on how texts shape knowledge-transfer and epistemic communities is vast and ever-growing. For a useful entry-point, see the contributions to Timothy Lenoir, *Inscribing Science: Scientific Texts and the Materiality of Communication* (Stanford, CA: Stanford University Press, 1998), esp. chs by Lenoir and Schaffer. Note also that my focus is accident neurosis and the various meanings attached to it by Miller. A broader study of malingering, the Welfare State and related disorders (e.g. compensation neurosis) awaits further research.

20 On temporal layers, see Manuel DeLanda, *A Thousand Years of Nonlinear History* (New York: Swerve Editions, 2000).

21 Miller, ‘Accident Neurosis: Lecture I’, 919 and 923. For background on the growth, from the Second World War, of accidental head injuries, see Alex R. Taylor, ‘Post-Concussional Sequelae’, *British Medical Journal*, 1967, 3, 70.

22 Miller, ‘Accident Neurosis: Lecture I’, 919. Miller would later add that around half of patients admitted to a head injury centre may be involved in litigation. See Miller, ‘Medicolegal Aspects of Head Injury’, 3.

23 Miller, ‘Accident Neurosis: Lecture I’, 919.

24
*Ibid*., 920.

25 Miller already had form with statistics, having published epidemiological studies of multiple sclerosis in the 1950s (see the bibliography in Lock and Windle, *Remembering Henry*, pp. 162–3). Furthermore, neurologists had routinely used statistics in broader studies of brain-functioning since at least the 1930s; they just tended to separate the compensation from the non-compensation cases, owing to the perceived psychological problems (e.g. cupidity) of the former. For instance, W. Ritchie Russell, ‘Cerebral Involvement in Head Injury: A Study Based on the Examination of Two Hundred Cases’, *Brain*, 1932, 55, 549–603.

26 E.g. Ramsay, ‘Nervous Disorder After Injury’.

27 Miller, ‘Accident Neurosis: Lecture I’, 920. Miller’s comments on the dearth of studies of traumatic neurosis were echoed in an editorial in the *British Medical Journal*. See no author, ‘Accident Neurosis’, *British Medical Journal*, 1961, 1, 1018.

28 Henry Miller and Niall Cartlidge, ‘Simulation and Malingering in Relation to Injuries of the Brain and Spinal Cord’, in Emanuel H. Feiring (ed.), *Brock’s Injuries of the Brain and Spinal Cord and Their Coverings* (New York: Springer, 1972), 643.

29 On Miller’s later complaints about terminology, and how this stymied effective comparison of different studies, see Henry Miller, ‘Posttraumatic Headache’, in P. J. Vinken and G. W. Bruyn (eds), *Headaches and Cranial Neuralgias: Handbook of Clinical Neurology, Vol. 5* (Wiley: New York, 1968), 179.

30 For praise of Miller’s use of ‘statistical support’, see no author, ‘Compensation for Cupidity?’; and J. A. Ryan, ‘Accident Neurosis’, *British Medical Journal*, 1961, 1, 1251.

31 Miller, ‘Accident Neurosis: Lecture I’, 921.

32 On the value of his statistical research in assessing prognosis, see Henry Miller and Gerald Stern, ‘The Long-Term Prognosis of Severe Head Injury’, *The Lancet*, 1965, 285, 225.

33
*Ibid.*, 227–8.

34 Miller, ‘Posttraumatic Headache’, 178.

35 Miller, ‘Mental After-Effects of Head Injury’, 259; and Henry Miller, ‘Headache’, *Transactions of the Ophthalmological Societies of the United Kingdom*, 1964, 84, 630.

36 Miller, ‘Accident Neurosis: Lecture I’, 920.

37 Lamentably, Miller did not explain which of his samples this group of 17 was drawn from, nor what their accidents were or how he had assessed them.

38 Similar sentiments can be found in J. A. Eddy, ‘Accident Neurosis’, *British Medical Journal*, 1961, 1, 1175.

39 Miller, ‘Accident Neurosis: Lecture I’, 922.; and Miller and Cartlidge, ‘Simulation and Malingering in Relation to Injuries of the Brain and Spinal Cord’, 642.

40 Miller, ‘Accident Neurosis: Lecture I’, 922.

41 Miller offered few solutions to this problem, however, and, like his peers, simply complained about legal delays and the need for better rehabilitation and reassurance. E.g. Henry Miller, ‘Compensation for Cupidity?’, *The Lancet*, 1961, 277, 1349–50.

42 Miller, ‘Accident Neurosis: Lecture I’, 923 and 925.

43
*Ibid.*, 925.

44 Miller, ‘Accident Neurosis: Lecture II’, 992. Miller would later estimate that around 15% of those injured, and about 30% of those who pursued a claim, were afflicted with neurosis. See Miller and Cartlidge, ‘Simulation and Malingering in Relation to Injuries of the Brain and Spinal Cord’, 645.

45 Miller, ‘Accident Neurosis: Lecture II’, 992.

46
*Ibid*., 993.

47 Henry Miller, ‘Mental After-Effects of Head Injury’, *Proceedings of the Royal Society of Medicine*, 1966, 59, 259; Miller and Cartlidge, ‘Simulation and Malingering in Relation to Injuries of the Brain and Spinal Cord’, 638 and *passim*.

48 C.f., the complaint that Miller’s intervention had not received enough attention in Frewen Moor, ‘Profitable Neurosis’, *British Medical Journal*, 1961, 1, 1459.

49 This was later reported in Miller, ‘After-Effects of Head Injury’.

50 E.g. R. N. Wilson, ‘Compensation for Cupidity?’, *The Lancet*, 1961, 278, 155; Ryan, ‘Accident Neurosis’, 1251; Moor, ‘Profitable Neurosis’, 1459. There were only a few critical responses to Miller, such as Michael Kelly, ‘Accident Neurosis’, *British Medical Journal*, 1961, 2, 110; and Martin F. Bethell, ‘Accident Neurosis’, *The Times*, 26 April 1961, 15.

51 See W. A. Reynard, ‘Compensation for Cupidity?’, *The Lancet*, 1961, 278, 52; S. H. F. Howard, ‘Compensation for Cupidity?’, *The Lancet*, 1961, 277, 1224.; and no author, ‘Accident Neurosis’.

52 E.g. J. N. Aston, ‘Settlement of Compensation’, *British Medical Journal*, 1961, 1, 1604; and no author, ‘Compensation for Cupidity?’, *The Lancet*, 1961, 277, 1099–100.

53 The *British Medical Journal* questioned whether Miller properly appreciated the need for more psychiatrists in the treatment of accident-sequelae, however. See no author, ‘Accident Neurosis’.

54 See no author, ‘Compensation for Cupidity?’.

55 E.g. Donald C. Norris, ‘Personal Injury and the Law’, *The Medico-Legal Journal*, 1962, 30, 65.; and Neustatter, ‘Psychiatric Disorders in Compensation Cases’, 166.

56 Note my emphasis on *explicit*. Although he did not reference Miller, an article by Charles Symonds in 1962 was interpreted as an implied challenge in no author, ‘Post-Concussional Syndrome’, *British Medical Journal*, 1967, 3, 61–2.

57 Note that Taylor separately argued for both, though it was the former that he was better known for. Taylor, ‘Post-Concussional Sequelae’; c.f., A. R. Taylor and T. K. Bell, ‘Slowing of Cerebral Circulation after Concussional Head Injury: A Controlled Trial’, *The Lancet*, 1966, 288, 178–80.

58 Taylor, ‘Post-Concussional Sequelae’, 68.

59 The 670 patients had suffered penetrating head injuries during the War, which were then followed up by neurologists at Oxford around five years later through questionnaires, psychological tests and, occasionally, re-examinations. See Michael Salzberg, ‘A Pioneer of Neuropsychiatry: Professor Alwyn Lishman’, *Australian and New Zealand Journal of Psychiatry*, 2004, 38, 5–6.

60 Indeed, an editorial in the *British Medical Journal* pondered whether physicians were actually studying the same syndrome. See no author, ‘Post-Concussional Syndrome’, *British Medical Journal*, 1967, 3, 62.

61 W. A. Lishman, ‘Psychiatric Disability after Head Injury: The Significance of Brain Damage’, *Proceedings of the Royal Society of Medicine*, 1966, 59, 261–6.

62 This article focuses on the responses to Miller in the British medical literature. For international responses, see the brief overview in Harold Merskey, ‘Psychiatry and the Cervical Sprain Syndrome’, *Canadian Medical Association Journal*, 1984, 130, 1119–21.

63 See Trethowan’s comments in Lock and Windle (eds), *Remembering Henry*, 90. Somewhat more euphemistically, Miller was accused of ‘rather overstat[ing] his case’ by one close colleague in Lord Walton, *The Spice of Life: From Northumbria to World Neurology* (London and New York: Royal Society of Medicine Services, 1993), 277. I am grateful to Stephen T. Casper for directing me to this reference.

64 W. H. Trethowan, ‘Rehabilitation of the Brain Injured: The Psychiatric Angle’, *Proceedings of the Royal Society of Medicine*, 1970, 63, 34.

65 As Gruneberg explained: ‘Miller’s contribution has stimulated critical reconsideration of the whole complex of related problems. Other work in this field is critical by implication, even without direct reference to Miller’s views.’ See R. Gruneberg, ‘The Concept of Accident Neurosis’, *Injury*, 1970, 1, 210–11.

66 W. A. Lishman, ‘The Psychiatric Sequelae of Head Injury: A Review’, *Psychological Medicine*, 1973, 3, 305–6 and *passim*.

67 Note the shift within this literature, in which the malingerer was to be found just as much within the National Health Service as within medico-legal practice. See J. L. Reed, ‘Disorders of Conscious Awareness: Compensation Neurosis and Munchausen Syndrome’, *British Journal of Hospital Medicine*, 1978, 19, 314–17; and J. M. Naish, ‘Problems of Deception in Medical Practice’, *The Lancet*, 1979, 314, 139–42.

68 E.g. D. W. Kay, T. A. Kerr and L. P. Gassman, ‘Brain Trauma and the Postconcussional Syndrome’, *The Lancet*, 1971, 298, 1052–5.

69 For background on Zangwill’s wartime research, see Alan F. Collins, ‘An Intimate Connection: Oliver Zangwill and the Emergence of Neuropsychology in Britain’, *History of Psychology*, 2006, 9, 89–112.

70 O. L. Zangwill, ‘Mental Sequelae of Head Injury’, *Proceedings of the Royal Society of Medicine*, 1966, 59, 266.

71 Frederick Lawton, ‘A Judicial View of Traumatic Neurosis’, *The Medico-Legal Journal*, 1978, 47, 9; the comments of Sir George Waller in Lock and Windle (eds), *Remembering Henry*, 94–5; and Harvey Teff, *Causing Psychiatric and Emotional Harm: Reshaping the Boundaries of Legal Liability* (Oxford and Portland, OR: Hart Publishing, 2009), 146–7.

72 For references to Miller’s statistics, see Lawton, ‘A Judicial View of Traumatic Neurosis’, 7 and 15; Reed, ‘Disorders of Conscious Awareness’, 317; and W. H. West, ‘Neurosis After Accidents’, *The Times*, 20 April 1961, 13.

73 E.g. see the comments on the unrepresentativeness of earlier studies (including Miller’s) in Jennifer H. Steadman and J. G. Graham, ‘Head Injuries: An Analysis and Follow-Up Study’, *Proceedings of the Royal Society of Medicine*, 1970, 63, 23 and 27.

74 E.g. the statistical study in H. Merskey and J. M. Woodforde, ‘Psychiatric Sequelae of Minor Head Injury’, *Brain*, 1972, 95, 521–8.

75 R. Kelly, ‘The Post-Traumatic Syndrome’, *Pahlavi Medical Journal*, 1972, 3, 530–1.

76 C.f., the comments of Sir George Waller in Lock and Windle, *Remembering Henry*, 93–5. He stated that Miller’s medico-legal practice was broad (encompassing both referrals from insurance-companies and trade-unions), and that Miller was indifferent to those whom he received instructions from.

77 R. Kelly, ‘The Post-Traumatic Syndrome: An Iatrogenic Disease’, *Forensic Science*, 1975, 6, 24; and Kelly, ‘The Post-Traumatic Syndrome’, 545, 538 and 540.

78 Kelly, ‘The Post-Traumatic Syndrome: An Iatrogenic Disease’, 23.

79 Kelly, ‘The Post-Traumatic Syndrome’, 532–3.

80 An overview of which is available in Kelly, ‘The Post-Traumatic Syndrome: An Iatrogenic Disease’, 17–19.

81 Kelly, ‘The Post-Traumatic Syndrome’, 531, 533–4 and 537–8.

82
*Ibid*., 545.

83 This evidence was earlier studies like Taylor’s in 1967. See Reginald Kelly, ‘The Post-Traumatic Syndrome’, *Journal of the Royal Society of Medicine*, 1981, 74, 242–5.

84 Reginald Kelly and Norman B. Smith, ‘Post-Traumatic Syndrome: Another Myth Discredited’, *Journal of the Royal Society of Medicine*, 1981, 74, 275–7.

85 Reginald Kelly, ‘Post-Traumatic Syndrome’, *Journal of the Royal Society of Medicine*, 1981, 74, 630–1.

86 For Miller’s publications around this time, see Lock and Windle (eds), *Remembering Henry*, 164–6.

87 See William H. Rutherford, John D. Merrett and John R. McDonald, ‘Sequelae of Concussion Caused by Minor Head Injuries’, *The Lancet*, 1977, 309, 1–4.; and W. H. Rutherford, J. D. Merrett and J. R. McDonald, ‘Symptoms at One Year Following Concussion From Minor had Injuries’, *Injury*, 1979, 10, 225–30. On the influence of Miller and Kelly, see the opening paragraphs of *ibid*.

88 Kelly, ‘The Post-Traumatic Syndrome’, 535.

89 E.g. Rutherford et al., ‘Sequelae of Concussion Caused by Minor Head Injuries’, 4, en. 1 and 2.

90 J. E. Woodyard, ‘Injury, Compensation Claims and Prognosis: Part I’, *Occupational Medicine*, 1980, 30, no2–5; and J. E. Woodyard, ‘Injury, Compensation Claims and Prognosis: Part II’, *Occupational Medicine*, 1980, 30, 57–60.

91 Woodyard concluded from this study that settlement did not guarantee the remission of symptoms, a point that had already been developed in numerous other studies. See J. E. Woodyard, ‘Diagnosis and Prognosis in Compensation Claims’, *Annals of the Royal College of Surgeons of England*, 1982, 64, 191–4.

92 Not all psychiatrists used statistics in their follow-up studies, however. Some still relied on case histories, as in A. C. White, D. Armstrong and D. Rowan, ‘Compensation Psychosis’, *The British Journal of Psychiatry*, 1987, 150, 692–4. On the growing use of statistics by psychiatrists, albeit in the American context, see Young, *The Harmony of Illusions*, esp. ch. 3.

93 Michael J. Tarsh and Claire Royston, ‘A Follow-Up Study of Accident Neurosis’, *The British Journal of Psychiatry*, 1985, 146, 18. C.f., Miller’s claim that he was not the first to undertake a follow-up study in Miller, ‘Mental After-Effects of Head Injury’, 258.

94 E.g. W. W. McKinlay, D. N. Brooks and M. R. Bond, ‘Post-Concussional Symptoms, Financial Compensation and Outcome of Severe Blunt Head Injury’, *Journal of Neurology, Neurosurgery and Psychiatry*, 1983, 46, 1084–91.

95 On Miller’s influence, see Robert I. Cohen and Jeremy M. Pfeffer, ‘Accident Neurosis Revisited’, *Medicine, Science and the Law*, 1987, 27, 177; and White et al., ‘Compensation Psychosis’, 692.

96 As noted in V. E. Weighill, ‘Compensation Neurosis: A Review of the Literature’, *Journal of Psychosomatic Research*, 1983, 27, 98–103.

97 For explicit references to Miller’s influence, see George W. Fenton, ‘The Postconcussional Syndrome: New Insights’, *Journal of the Royal Society of Medicine*, 1994, 87, 499; Richard Mayou, ‘Accident Neurosis Revisited’, *The British Journal of Psychiatry*, 1996, 168, 399; and K. Mealy, H. Brennan and G. C. Fenelon, ‘Early Mobilization of Acute Whiplash Injuries’, *British Medical Journal (Clinical Research Ed.)*, 1986, 292, 657.

98 E.g. Malcolm I. V. Jayson, ‘Trauma, Back Pain, Malingering, and Compensation’, *British Medical Journal*, 1992, 305, 7; and Mealy et al., ‘Early Mobilization of Acute Whiplash Injuries’, 656. On the simplification of references over time, see Susan E. Cozzens, ‘Comparing the Sciences: Citation Context Analysis of Papers From Neuropharmacology and the Sociology of Science’, *Social Studies of Science*, 1985, 15,142, 146–7.

99 For instance, R. R. Jacobson, ‘The Post-Concussional Syndrome: Physiogenesis and Malingering. An Integrative Model’, *Journal of Psychosomatic Research*, 1995, 39, 684; or Mayou, ‘Accident Neurosis Revisited’, 399.

100 Ruth Leys, *Trauma: A Genealogy* (Chicago and London: The University of Chicago Press, 2000), 6.

101 On the models of time and causation bound up in different theories of trauma, see Young and Breslau, ‘What Is PTSD?’.

## Notes

Ryan Ross completed his PhD in 2017 at the Centre for the History of Emotions, Queen Mary, University of London. His thesis, ‘Accidents and Their Medico-Legal Sequelae in Twentieth-Century Britain: Time, Trauma and Writing History’, examined how the medical profession framed the medico-legal sequelae of traumatic accidents in Britain, *c.* 1930 to 1990—specifically, how doctors diagnosed, examined and conceptualised disorders like traumatic neurosis, post-concussional syndrome and whiplash. He continues to teach at Queen Mary, and blogs at www.timetraumahistory.wordpress.com. You can follow Ryan on Twitter @Ryan_RR_Ross.

